# The Relationship between Thermoregulation and REM Sleep Behaviour Disorder in Parkinson’s Disease

**DOI:** 10.1371/journal.pone.0072661

**Published:** 2013-08-21

**Authors:** George Zhong, Samuel Bolitho, Ronald Grunstein, Sharon Linda Naismith, Simon John Geoffrey Lewis

**Affiliations:** 1 Parkinson’s Disease Research Clinic, Brain and Mind Research Institute, The University of Sydney, Sydney, New South Wales, Australia; 2 Sleep and Circadian Research Group, Woolcock Institute of Medical Research, The University of Sydney, Sydney, New South Wales, Australia; Centre Hospitalier de l'Université Laval, Canada

## Abstract

**Background:**

This study explored the relationship between symptoms of rapid eye movement sleep behaviour disorder, thermoregulation and sleep in Parkinson’s Disease.

**Methods:**

The study group comprised 12 patients with Parkinson’s Disease and 11 healthy age-matched controls. We investigated markers of thermoregulation (core-body temperature profile), circadian rhythm (locomotor actigraphy) and sleep (polysomnography).

**Results:**

The mesor (the mean value around which the core temperature rhythm oscillates) of the core-body temperature in patients with Parkinson’s Disease was significantly lower than that of controls. In addition, the nocturnal fall in CBT (the difference between the mesor and the nadir temperature) was also significantly reduced in PD patients relative to controls. Furthermore, in patients the reduction in the amplitude of their core-body temperature profile was strongly correlated with the severity of self-reported rapid eye movement sleep behaviour disorder symptom, reduction in the percentage of REM sleep and prolonged sleep latency. By contrast, these disturbances of thermoregulation and sleep architecture were not found in controls and were not related to other markers of circadian rhythm or times of sleep onset and offset.

**Conclusions:**

These findings suggest that the brainstem pathology associated with disruption of thermoregulation in Parkinson’s disease may also contribute to rapid eye movement sleep behavioural disorder. It is possible that detailed analysis of the core-body temperature profile in at risk populations such as those patients with idiopathic rapid eye movement sleep behaviour disorder might help identify those who are at high risk of transitioning to Parkinson’s Disease.

## Introduction

Traditionally Parkinson’s disease (PD) has been characterised by its constellation of motor symptoms, but non-motor symptoms such as disturbances of sleep, circadian rhythm and thermoregulation have been increasingly recognised[1], [2], [3], [4], [5], [6]. Rapid eye movement (REM) sleep behaviour disorder (RBD) is reported in up to 60% of PD patients[7]. This symptom is characterised by loss of muscle atonia during REM sleep, which results in violent nocturnal dream enactments[8]. There is increasing evidence to suggest that RBD can actually precede the diagnosis of PD by many years[9], [10] and in one series of patients presenting with idiopathic RBD, nearly 20% developed neurodegenerative disease over a 5-year period, with the majority developing PD[11].

The pathogenesis of RBD in PD is thought to involve pontine pathology within the sublaterodorsal nuclei as well as descending projections to the medullary magnocellular reticular formation, which are commonly regarded as REM-on nuclei of the ultradian system [12]. Furthermore, the ultradian system is intimately related through extensive neural connections to the circadian system, which regulates the 24-hour cycle of sleep and wakefulness, and the homeostatic system, which regulates the onset and offset of sleep. For detailed reviews of the neuroanatomy and function of the circadian-homeostatic-ultradian system, see [13], [14]. Given that the circadian-homeostatic-ultradian and thermoregulatory systems all have critical components that reside within the pons and medulla, it might be anticipated that the pathology underlying RBD would also impact on these systems [15], [16], [17], [18].

Currently, there is a paucity of literature examining disruptions to thermoregulation in Parkinson’s disease[19], [20], [21]. Existing studies have been limited by small sample sizes, the absence of a control group and examining core body temperature profiles only as an adjunct to other primary variables. Furthermore, we are not aware of any studies in the literature to date that directly examine the relationship between thermoregulation and sleep architecture in Parkinson’s Disease. In the present study, we examined differences in thermoregulation between patients with Parkinson’s disease and controls as well as its correlations with markers of circadian rhythm, sleep and self-reported symptoms of RBD using an array of established techniques including core-body temperature (CBT) profiling, locomotor actigraphy and polysomnography (PSG). We hypothesised that patients with Parkinson’s disease would show differences in their core-body temperature profile when compared to controls. Furthermore, we hypothesised that this pattern of disruption may be further associated with the symptoms of RBD due to underlying pathology potentially affecting overlapping neural circuitry.

## Methods

### Ethics Statement

This research was approved by the Human Research Ethics Committee of the University of Sydney (approval number 11105). Written informed consent was obtained from all participants. All participants had no significant cognitive impairment on structured assessment and were deemed independently by an experienced neurologist (SJGL) and neuropsychologist (SLN) to have the capacity to consent.

### Participants

Twelve patients with PD (8 males, 4 females) satisfying UK PDS Brain Bank criteria[22] were recruited from the PD Research Clinic at the Brain and Mind Research Institute, University of Sydney. Eleven controls (6 males, 5 females) with no sleep complaints were recruited from the community, through advertisement.

*Exclusion criteria* were: history of stroke; neurological disorder (other than PD); head injury with loss of consciousness ≥ 30-minutes; medical condition known to affect cognition (e.g. cancer); psychiatric illness; Mini-Mental State Examination Score (MMSE)[23] < 24 and/or diagnosis of dementia; shiftworkers; transmeridian travel within the prior 60-days, use of medication known to affect sleep and/or melatonin secretion including beta-blockers, lithium, and benzodiazepines. Patients taking sedative hypnotics were requested to abstain for two-weeks prior to sleep assessment.

### Clinical Assessments

Patients underwent neurological examination conducted in their ‘on’ state and levodopa dose equivalents were calculated for dopaminergic medications[24]. Disease stage was rated on the Hoehn and Yahr scale (H&Y)[25] and motor severity was rated on the Unified Parkinson’s Disease Rating Scale section III (UPDRS-III)[26]. Disease duration was calculated from age at disease diagnosis and depressive symptoms were self-rated using the Beck Depression Inventory-II (BDI-II, scores > 20 are indicative of at least ‘moderate’ mood disturbance)[27]. The REM Sleep Behavior Disorder Screening Questionnaire (RBDSQ)[28] was used to assess RBD symptoms (score range between 0 and 13, inclusive, scores > 5 are suggestive of significant RBD[29]). All controls scored zero on the RBDSQ.

### Locomotor Actigraphy

Participants’ average sleep duration for 14-nights prior to commencing the in-laboratory portion of the experimental protocol was recorded using actigraphy (*Actiwatch Spectrum, Minimitter-Respironics, OR*) in accordance with previously published protocols[30], [31]. Rest interval measures were times of rest onset and rest offset, both recorded in 24-hour clock time.

### Laboratory assessment of sleep and thermoregulatory systems

All volunteers underwent PSG and CBT recording in the Chronobiology & Sleep Laboratory, Brain & Mind Research Institute, University of Sydney. Participants attended the laboratory seven hours prior to their habitual sleep onset time as identified by actigraphy and confirmed by sleep diary. While in the laboratory, participants were physiologically and behaviourally monitored under controlled conditions at all times, with fixed light levels (< 50 lx during waking; < 1 lx during scheduled sleep periods) and ambient temperature (24±1°C). Participants were asked to refrain from alcohol or caffeine consumption for the preceding 24-hour period.

### Polysomnography (PSG)

Participants were monitored on two consecutive nights (*Compumedics Siesta, Australia*). Night 1 was considered an adaptation night and included pressure flow monitoring to detect significant obstructive sleep apnoea. Sleep architecture variables were collected on night 2 with a standardised research PSG montage (electroencephalogram, electrooculogram, electromyogram)[32] and sleep stages were visually scored using established criteria[33], [34]. Outcome variables from the PSG assessment used for analyses included times of sleep onset and offset (24-hour clock time), sleep and REM sleep latencies (defined as the time between light off and PSG demonstrated sleep and REM onset, respectively), sleep efficiency (calculated by [duration of sleep on PSG/duration in bed] * 100%), % slow wave sleep and % REM sleep. The EMG trace of the PSG was analysed for the presence of REM sleep without atonia (RWA), which according to International Classification of Sleep Disorders (ICSD)-2 forms part of the diagnostic criteria for RBD[35].

### Core-body temperature (CBT) profiling

**Figure 1:**
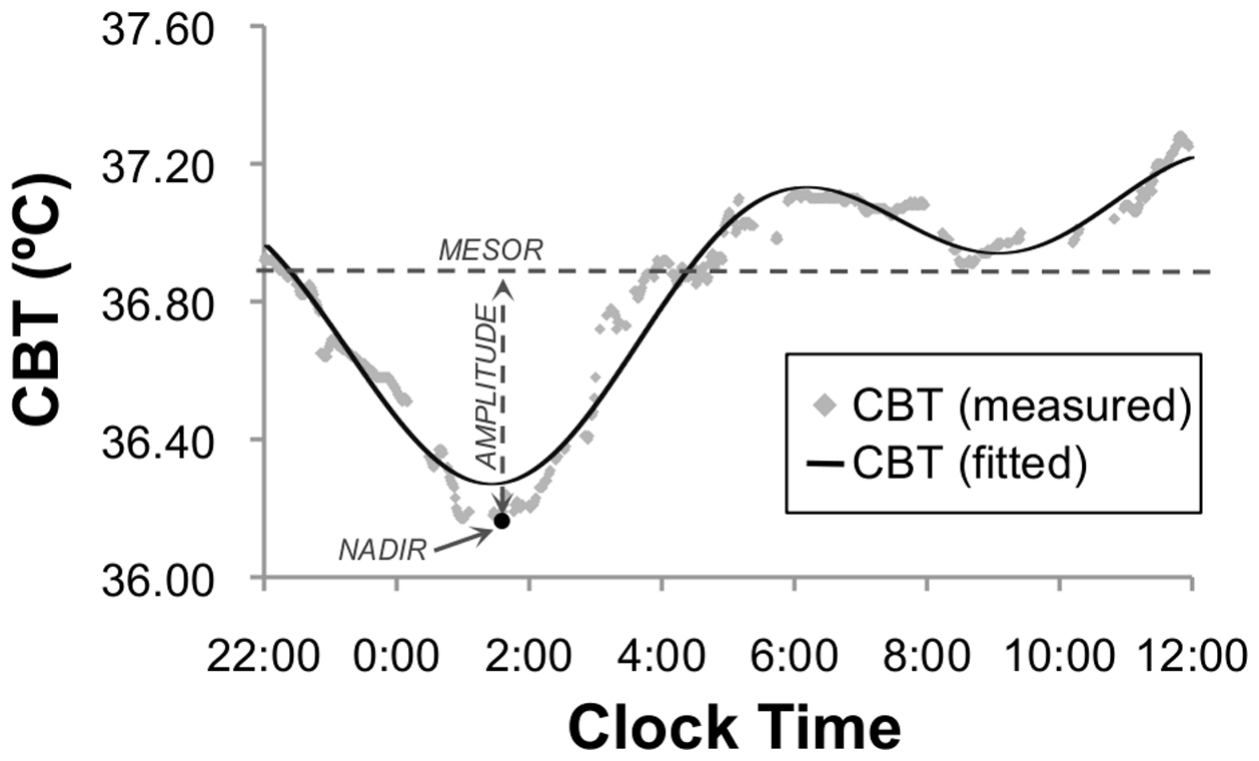
Schematic diagram showing a typical nocturnal core-body temperature (CBT) profile. The mesor is defined as the average value around which the CBT fitting oscillates. The nadir is defined as the lowest temperature during sleep. The amplitude is defined as the difference between the mesor and the nadir.

The CBT profile (Figure 1) was recorded for 24-hours with an ingestible capsule sensor and data was transmitted wirelessly to a recording unit (*VitalSense, Minimitter-Respironics, OR*). During sleep, CBT was recorded concurrently with PSG to allow for accurate comparison with key sleep parameters, such as sleep onset, offset and architecture. Analysis of the CBT profile involved least squares fitting with two spherical harmonics in order to extract the temperature mesor (the mean value around which the core temperature rhythm oscillates), in line with the cosinor method[36]. Time and temperature of the nocturnal nadir, defined as the point of lowest CBT during sleep, were also extracted. All CBT analyses were conducted in MATLAB R2012a, Version 7.14, Natick, Massachusetts: The Mathworks Inc.

### Statistical analysis

All statistical analyses were performed using SPSS for Macintosh, Rel. 18.0.0 Chicago: SPSS Inc. Unless otherwise stated, statistical tests for significance between groups were conducted using the independent Student’s t-test. Where there was no homogeneity of variance, or when data was not normally distributed, t-test with unequal variances or Mann-Whitney U tests were used respectively. The Spearman correlation coefficient was used for all correlations. All results were reported in the format of mean ± standard deviation. All analyses were two-tailed and employed an alpha level of 0.05.

## Results

### Demographics

Table 1 summarises the demographic profile and results of the PSG, actigraphy and CBT measurements for PD patients and controls. There were no significant differences in age, gender composition, MMSE or BDI-II scores between the PD and control groups.

**Table 1:** Demographic, clinical assessment, circadian, sleep and temperature data (mean ± SD) for PD patients and controls.

Variable	PD	Control	p-value
N	12	11	–
Age (years)	62.2±8.9	66.6±7.1	0.20^†^
Gender (% male)	67	55	0.87^#^
***Clinical assessment***			
MMSE	28.7±1.6	29.0±1.2	0.82^†^
BDI-II	4.8±2.5	6.4±7.8	0.69^†^
RBDSQ	5.0±4.6	0.0±0.0	–
PD duration (years)	2.8±3.6	–	–
H&Y (stage)	1.8±0.6	–	–
Levodopa dose equivalent (mg)	276±336	–	–
UPDRS-III	23.2±6.6	–	–
***Polysomnography***			
Sleep onset (time)	22:31±0:44	23:14±1:03	0.13^†^
Sleep offset (time)	06:24±0:43	06:53±1:10	0.13^†^
Sleep onset latency (min)	13.5±10.2	19.6±12.7	0.23
REM sleep latency (min)	82.4±45.1	115.6±89.0	0.52^†^
Sleep efficiency (%)	74.8±12.4	72.6±8.0	0.62
% Slow wave sleep	15.4±9.2	12.4±8.9	0.42
% REM sleep	20.0±7.0	20.6±7.0	0.82
***Actigraphy***			
Rest onset (time)	22:26±0:59	23:27±1:09	0.08^†^
Rest offset (time)	07:00±0:38	07:34±1:11	0.23^†^
***Core-body temperature***			
Nadir (time)	01:54±2:04	02:30±2:05	0.50
Nadir temperature (°C)	36.13±0.25	36.18±0.23	0.42^†^
Mesor (°C)	36.63±0.24	36.87±0.17	0.02^†^ *
Amplitude (°C)	0.50±0.16	0.69±0.29	0.04^†^ *

* p<0.05; Data tested with students t-test except: ^#^ the χ^2^ statistic with Yates’ correction was used to compare gender composition between groups; ^†^ the Mann-Whitney U test was used for data with non-normal distributions.

MMSE, Mini-Mental State Examination; BDI-II, Beck Depression Inventory II; H&Y, Hoehn and Yahr scale; UPDRS-III, Unified Parkinson’s disease rating scale part III; RBDSQ, REM Sleep Behaviour Disorder Screening Questionnaire.

### Actigraphy and PSG

Table 1 shows that for the two weeks prior to laboratory assessment, PD patients had slightly earlier rest onset times on actigraphy than controls (U = 95, p = 0.08), a trend also observed in the PSG sleep onset time (U = 91, p = 0.13) but neither reached statistical significance. There was no significant difference in the time of rest/sleep offset on actigraphy or PSG, respectively. There was no significant difference in sleep architecture between PD patients and controls on PSG.

### Core-body temperature profile

As shown in Table 1, the temperature mesor for PD patients was significantly lower than that of controls (U = 104, p = 0.02). The nocturnal CBT amplitude, calculated using the difference between the nadir temperature and the mesor, for PD patients was also significantly reduced relative to controls (U = 100, p = 0.04). There was no significant difference between PD patients and controls with respect to the time and temperature of the nocturnal nadir, defined as the point where the CBT reaches its minimum during sleep.

### Correlation of CBT profile with RBDSQ, circadian and sleep variables in PD

**Figure 2:**
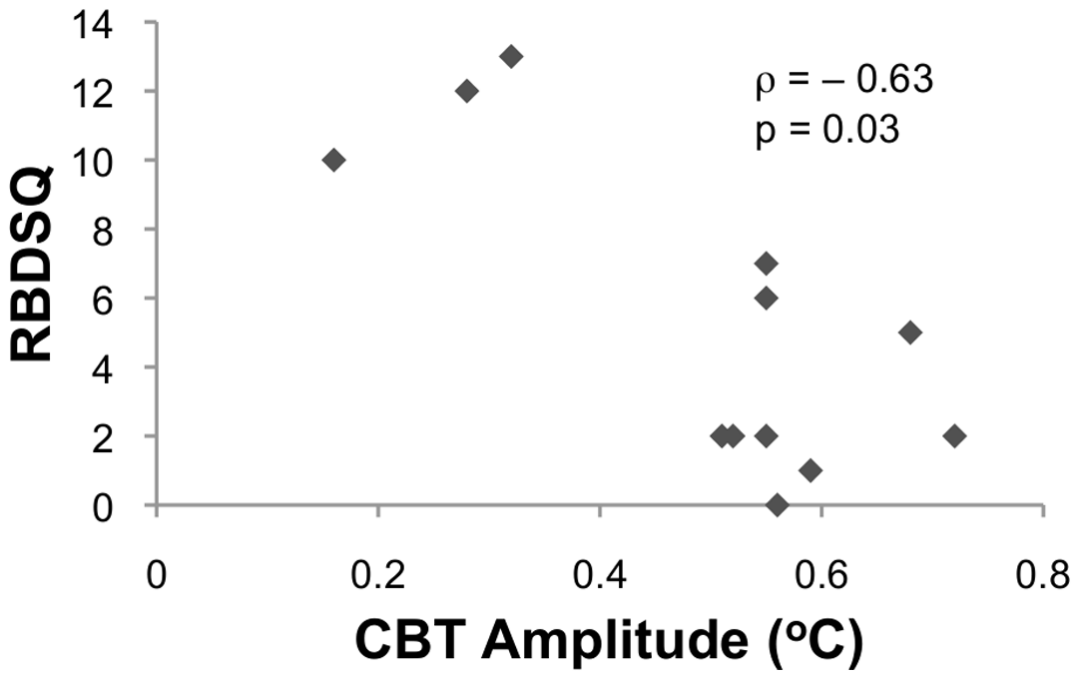
Scatter plot demonstrating the strong negative correlation between self-reported symptoms of the nocturnal CBT amplitude and REM Sleep Behaviour Disorder Questionnaire (RBDSQ) score.

As shown in Figure 2, reduced amplitude of the CBT profile was strongly correlated with severity of self-reported RBD symptoms on RBDSQ among PD patients (rho = −0.63, p = 0.03). Recent work has reported that a score of 6 on the RBDSQ represents the best cut-off value for detecting RBD in PD[29]. Using this approach revealed that five PD patients had self-reported RBD (i.e., with RBDSQ score > 5), of which four were found to demonstrate RWA on their PSG analysis. None of the seven PD patients without self-reported RBD (i.e., with RBDSQ score ≤ 5) demonstrated RWA on their PSG analysis. The five patients who self-reported RBD were found to have significantly reduced CBT profile amplitude compared to the seven patients without RBD (0.37±0.17 vs 0.59±0.08, t = 2.6, p = 0.04).

Reduced nocturnal CBT amplitude was also associated with significant reduction in the percentage of REM sleep (rho = 0.71, p = 0.01) and increase in sleep onset latency (rho = −0.61, p = 0.03) among PD patients but not controls. The nocturnal CBT amplitude did not correlate significantly with age, disease duration, disease severity (H&Y or UPDRS-III), levodopa dose equivalents, mood (BDI-II) or any other PSG or actigraphy parameters measured.

## Discussion

**Figure 3:**
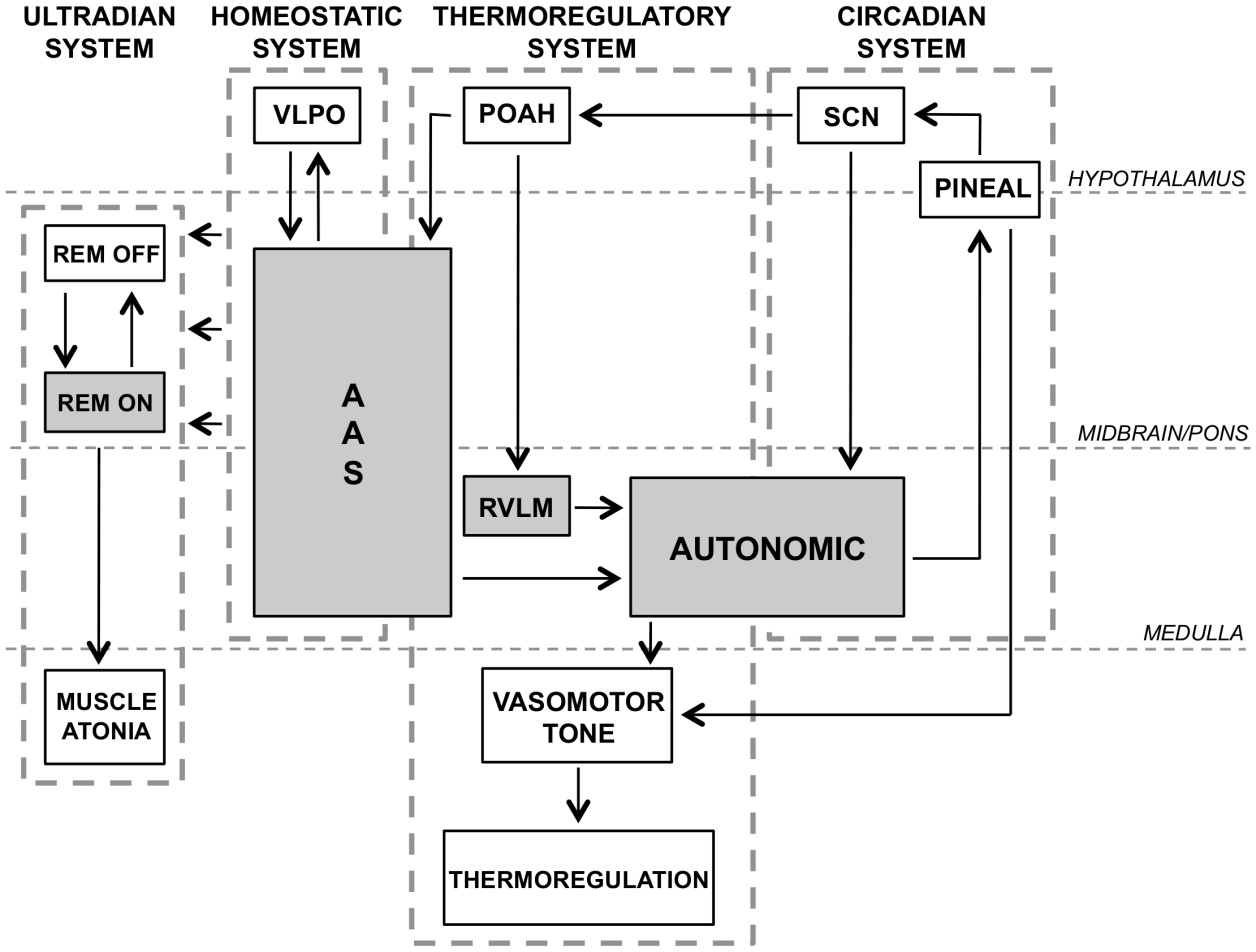
Simplified schematic diagram highlighting the neural circuitry of the circadian-homeostatic-ultradian system and the functional neuroanatomical overlap with the thermoregulatory system. Shaded regions denote sites of pathological damage in PD that have been demonstrated in previous neuropathological studies. KEYS: Ventrolateral preoptic nucleus (VLPO); ascending arousal system (AAS); preoptic nuclei of anterior hypothalamus (POAH); rostral ventrolateral medulla (RVLM); medullary autonomic region (AUTONOMIC); suprachiasmatic nuclei (SCN); pineal gland (PINEAL).

In the present study, we found significant differences between the CBT profiles of PD patients as compared to that of controls, suggesting that the neural circuitry controlling thermoregulation is disrupted in PD. Furthermore, this is the first study to our knowledge to demonstrate a relationship between the CBT profile and self-reported RBD symptoms in PD[37]. Specifically, the reduction in nocturnal CBT amplitudes was strongly correlated with the severity of self-reported RBD symptoms as well as reduction in sleep efficiency. This may be due to extensive functional overlap between the thermoregulatory system and circadian-homeostatic-ultradian system especially within the pons and medulla, as shown in Figure 3.

Indeed, previous neuropathological studies in humans have found Lewy body pathology affecting these key regions of overlap in the pons and medulla[38] that could potentially explain the thermoregulatory and sleep disturbances observed in the present study. The ascending arousal system (AAS), which is disrupted in PD[38] plays a significant role in the regulation of sleep initiation and maintenance. Furthermore, the AAS also influences sleep architecture via projections to the ultradian system and controls thermoregulation via projections to the medullary autonomic region[39], [40]. Thus, pathology within the AAS could explain the reduced amplitude of the CBT profile recorded in PD patients as compared to controls as well as the observed correlations with RBD severity and reduction in REM sleep. Neuropathology in this region may further explain the observed correlation between reduced nocturnal CBT amplitude with prolonged sleep latency, reflecting disruptions to the neural circuitry underlying sleep initiation.

The medullary autonomic region also plays a major role in thermoregulation via the modulation of peripheral vasomotor tone[17], [18] and this region has also been shown to exhibit Lewy body pathology in PD[38]. Furthermore, the medullary autonomic region is also responsible for relaying circadian signals from the master pacemaker, which resides in the suprachiasmatic nuclei (SCN) of the hypothalamus, to the pineal gland[41], which is responsible for melatonin secretion. Therefore, pathological damage to this region could also contribute to the observed reduction in nocturnal CBT amplitude as well as potential disruptions to the circadian rhythm and melatonin secretion patterns. Indeed, previous studies have shown that the melatonin secretion pattern is modified in PD relative to controls [21], though these patients had more advanced PD than those in the present study.

Although there was a trend towards circadian phase advancement in the PSG sleep onset, actigraphy rest onset and CBT nadir of PD patients relative to controls in the present study, these results did not reach statistical significance. This finding argues that the master pacemaker is probably devoid of significant pathology, which would be consistent with Braak’s caudorostral progression of Lewy body pathology in PD[38], whereby the medullary and pontine projections are predominantly affected with relative sparing of hypothalamic nuclei. In fact, previous neuropathological studies have demonstrated that the SCN is largely spared even in relatively advanced PD[42]. It may also be possible that the absence of statistical significance is due to small group sizes employed in the present study and that there is an element of internal desynchrony occurring between the circadian, sleep and thermoregulatory systems in PD. The latter may partly explain the therapeutic effect of melatonin in the treatment of RBD that has been reported in previous studies[43], [44], [45].

Finally, it should be noted that dopaminergic therapy may also influence the circadian CBT profile. Previous studies have found that higher doses of dopaminergic therapy may be associated with reduced circadian CBT amplitude[21]. Although, there was no significant correlation between the nocturnal CBT amplitude and levodopa dose equivalent in the present study, this may be due to the relatively low doses of dopaminergic therapy used or the lack of statistical power to demonstrate a relationship. Further studies may be warranted to explore this relationship.

Despite being the largest study to date examining the relationship between CBT profile and sleep in Parkinson’s disease, the present study is still clearly limited by small group sizes. Thus, detailed interpretation of the CBT profiles and its associations with other sleep and circadian markers should be made with caution. However, given their close relationship, it is possible that a more detailed analysis of the CBT profile in future studies may help extend our understanding of these disruptions. Furthermore, an appreciation of the CBT profile may represent a critical advance in our ability to identify those patients with idiopathic REM sleep behaviour disorder who are at higher risk of transitioning to PD.

## Conclusions

The present study shows significant differences in the nocturnal CBT profile of PD patients compared to that of controls. Furthermore, this is the first study to demonstrate a relationship between the nocturnal CBT profile and RBD symptoms in PD patients. The findings of this study suggest there are overlaps between neural circuitries regulating REM atonia, thermoregulation and wakefulness. As a result of this relationship, detailed analysis of CBT profiles in future studies could potentially shed light on the pathophysiology of RBD and its transition to PD.
